# Myopia and depressive symptoms among older Chinese adults

**DOI:** 10.1371/journal.pone.0177613

**Published:** 2017-05-12

**Authors:** Yin Wu, Qinghua Ma, Hong-Peng Sun, Yong Xu, Mei-E Niu, Chen-Wei Pan

**Affiliations:** 1 Department of Nursing, the First Affiliated Hospital of Soochow University, Suzhou, China; 2 The 3^rd^ People’s Hospital of Xiangcheng District, Suzhou, China; 3 School of Public Health, Medical College of Soochow University, Suzhou, China; National Eye Institute, UNITED STATES

## Abstract

**Purpose:**

Few population-based data support the hypothesis that refractive errors are associated with depressive symptoms. We aim to assess the impact of myopia on the risk of having depressive symptoms in a community-based cohort of elderly Chinese.

**Methods:**

A community-based cross-sectional study of 4611 Chinese adults aged 60 years or older was conducted. Depressive symptoms were measured using the 9-item Patient Health Questionnaire (PHQ-9) depression scale in 4597 adults. Refraction was determined by auto-refraction followed by subjective refraction. Myopia was defined as spherical equivalent (SE) < -0.50 diopters (D) and high myopia as SE < -6.00 D.

**Results:**

After adjusting for age, gender, education, lifestyle-related exposures, presenting visual acuity and age-related cataract, myopic adults were more likely to have any depressive symptoms compared with non-myopic ones (odds ratio = 1.39; 95% confidence interval 1.04, 1.92). There were no significant differences in the risk of having any depressive symptoms between those with and without high myopia. Myopia or high myopia was not associated with having moderate depressive symptoms. The impact of myopia on depressive symptoms was stronger in adults with no formal education compared with those with formal education.

**Conclusions:**

Myopia was related with the presence of depressive symptoms among older adults.

## Introduction

Myopia is a worldwide health problem[1], especially in Asians[2], and imposes heavy socioeconomic burdens on individuals, communities, and countries. Health economic studies have always shown that the medical cost of myopia far exceeded those of other major eye conditions such as retinal diseases (e.g. age-related macular degeneration and diabetic retinopathy), glaucoma, and is only secondary to age-related cataract.[3, 4] While the risk factors for myopia including both genetic and environmental ones have been described by numerous studies[5], its impacts have been less well defined. Previous studies have focused on the impact of myopia on other vision-threatening eye diseases[6], cognitive dysfunction[7], socioeconomic cost[8], and health-related quality of life[9]. However, little has been understood regarding its impact on people’s mental health such as depressive symptoms.

Depression is an chronic but often recurrent psychiatric disorder among older people and is associated with mortality[10] and comorbidities[11]. Previous study has shown that depression is an important mental health problem in ophthalmological practice but is often unrecognized or untreated[12]. Vision is the most important sensorial part of the information system for human beings. Visual loss might weaken the ability to perform routine activities in daily lives and can impair stable mental health. Visual impairment may also isolate individuals from communicating with friends and family. Therefore, untreated visual impairment may have tremendous negative psychological impacts. However, few population-based data support the hypothesis that myopia or other eye disorders are associated with the presence of depressive symptoms. In this study, we aim to assess the association of myopia with depressive symptoms in a community-based cohort of elderly Chinese people aged 60 years or older.

## Methods

### Study population

The Weitang Geriatric Diseases study was a community-based survey conducted in the Weitang town located in Suzhou, an urban metropolis in eastern China.[13, 14] The aim of the study was to estimate the patterns, predictors and burden of common health outcomes of older adults aged 60 years or older in eastern China. Based on official records, 6030 individuals aged 60 years or older resided in the town. Before the study, an invitation letter was sent to each family and nature of the study was explained in the letter. All the adults aged 60 years or older in the town were invited to participate in this study. An adult was considered “ineligible” to participate in this study if he or she had moved from the residing address, had not been living there for more than 6 months, or was deceased. Of the 6030 names listed in the official records, 5613 subjects were considered to be “eligible” to participate in this study. From August 2014 to February 2015, a total of 4611 elderly adults (82.1%) participated in this study.

The Weitang Geriatric Diseases study was conducted following the tenets of the Helsinki Declaration and was approved by the Institutional Review Board of Soochow University. The study methods were carried out in accordance with the approved guidelines. Informed consent was obtained from all study participants.

### Assessment of refractive errors

Objective refraction was measured using an autorefractor (Canon RK-5 Auto Ref- Keratometer, Canon Inc Ltd, Tokyo, Japan). Manual subjective refraction was then used to refine vision, using the results of the objective refraction as the starting point. In this study, refraction data were obtained from subjective refraction techniques. If subjective refraction was not available, autorefraction data were used instead. Spherical equivalent (SE) was defined as sphere plus half cylinder. Any myopia was defined as a SE of less than -0.5 diopter (D) while high myopia was defined as a SE of less than -6.0 D.

### Measurement of depressive symptoms

We used the 9-item Patient Health Questionnaire (PHQ-9) depression scale to assess the frequency of symptoms experienced and reported by the study participants within the past two weeks.[15] The PHQ-9 scores range from 0 to 27 and indicate the presence and severity of depressive symptoms, with scores of 5 and 10 being the cut-points for mild and moderate depression, respectively. The validity and reliability of the PHQ and its 9-item depression module to establish depressive diagnosis and grade severity have been documented in previous report.[16] In the present study, the severity of depressive symptoms was evaluated using 2 categories: any depressive symptoms (PHQ-9 score, 5–27) and any moderate depressive symptoms (PHQ-9 score, 10–27).

### Assessment of covariates

A risk factor questionnaire asking about the participants’ socioeconomic status, lifestyle-related factors, disease histories and medication intake was administered by trained research assistants. Diabetes mellitus was defined as fasting glucose levels of more than 7.0 mmol/L or physician diagnosis of diabetes and use of diabetic medications.[17] Hypertension was defined as systolic blood pressure of 140mmHg or more or diastolic blood pressure of 90mmHg or more, or use of antihypertensive medication. Slit-lamp examination (model SL-1E; Topcon) was performed for both eyes in each study participant and included a clinical grading of lens opacity using the Lens Opacities Classification System (LOCS) III.[18]

### Statistical analysis

As the correlation coefficient for SEs in the left and right eye was high and the results of analysis in both eyes were similar, only the results for right eye data were presented. Study participants with pervious cataract surgery in the right eye were excluded from analyses. Binary logistic regression models were fitted to estimate the associations of myopia or high myopia with any depression or any moderate depression and the effect estimate of odds ratio (OR) and its relative 95% confidence interval (CI) were calculated. For multivariate analysis, only age, gender, myopia and factors that were significantly different in univariate comparison (P<0.05) were retained in the model. The Akaike Information Criterion (AIC) was used to select influential explanatory variables and any explanatory variable would be excluded if it did not result in a better model fit. Interaction effects (different combination of the variables) between myopia and other risk factors on the depressive symptoms were determined using a likelihood ratio test. Statistical analyses were performed using SPSS 16.0 (SPSS Inc., Chicago, IL) and a P value of less than 0.05 indicated statistical significance.

## Results

**Fig 1:**
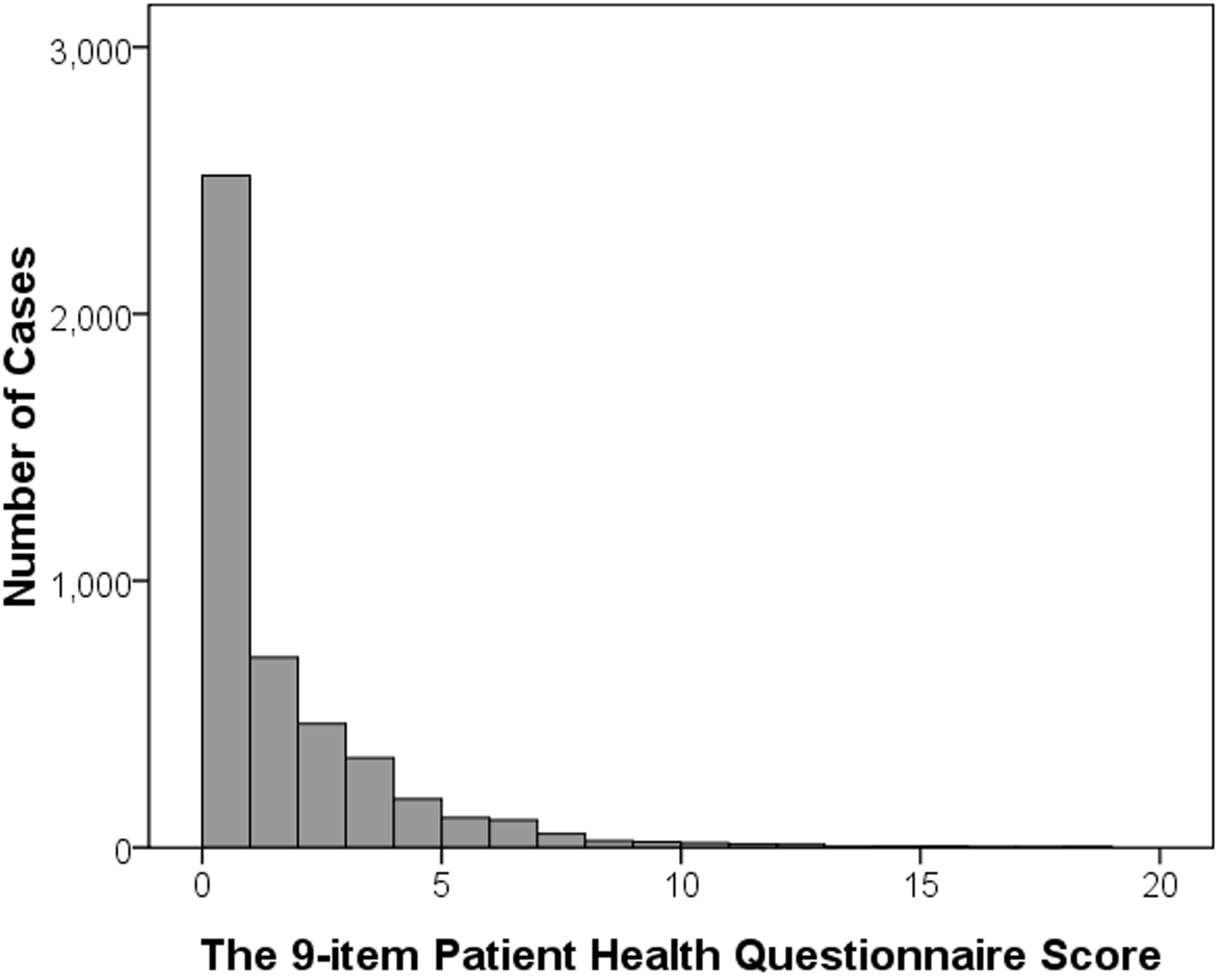
Distribution of PHQ-9 scores.

**Fig 2:**
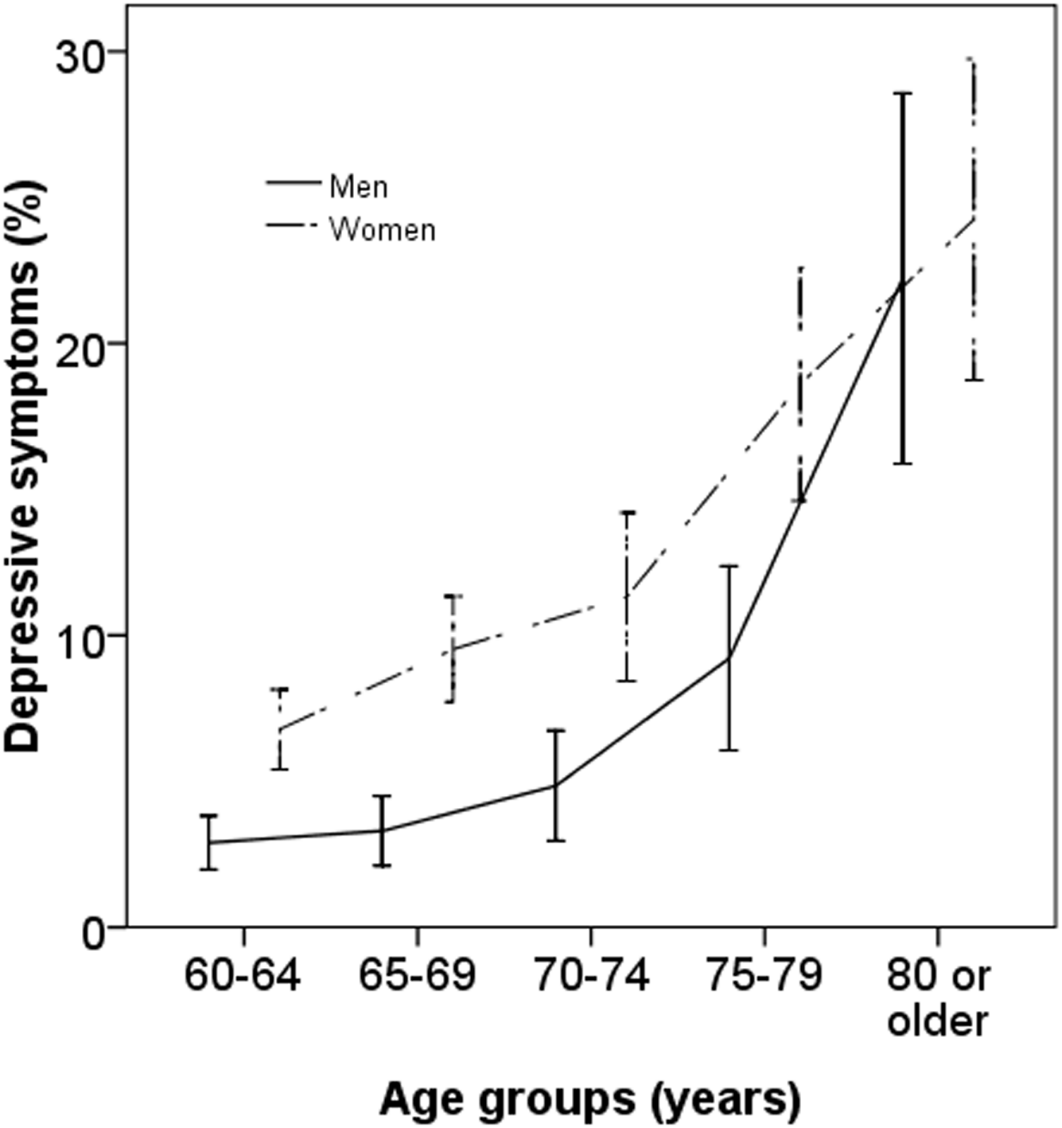
Age-specific prevalence of depression by gender.

**Fig 3:**
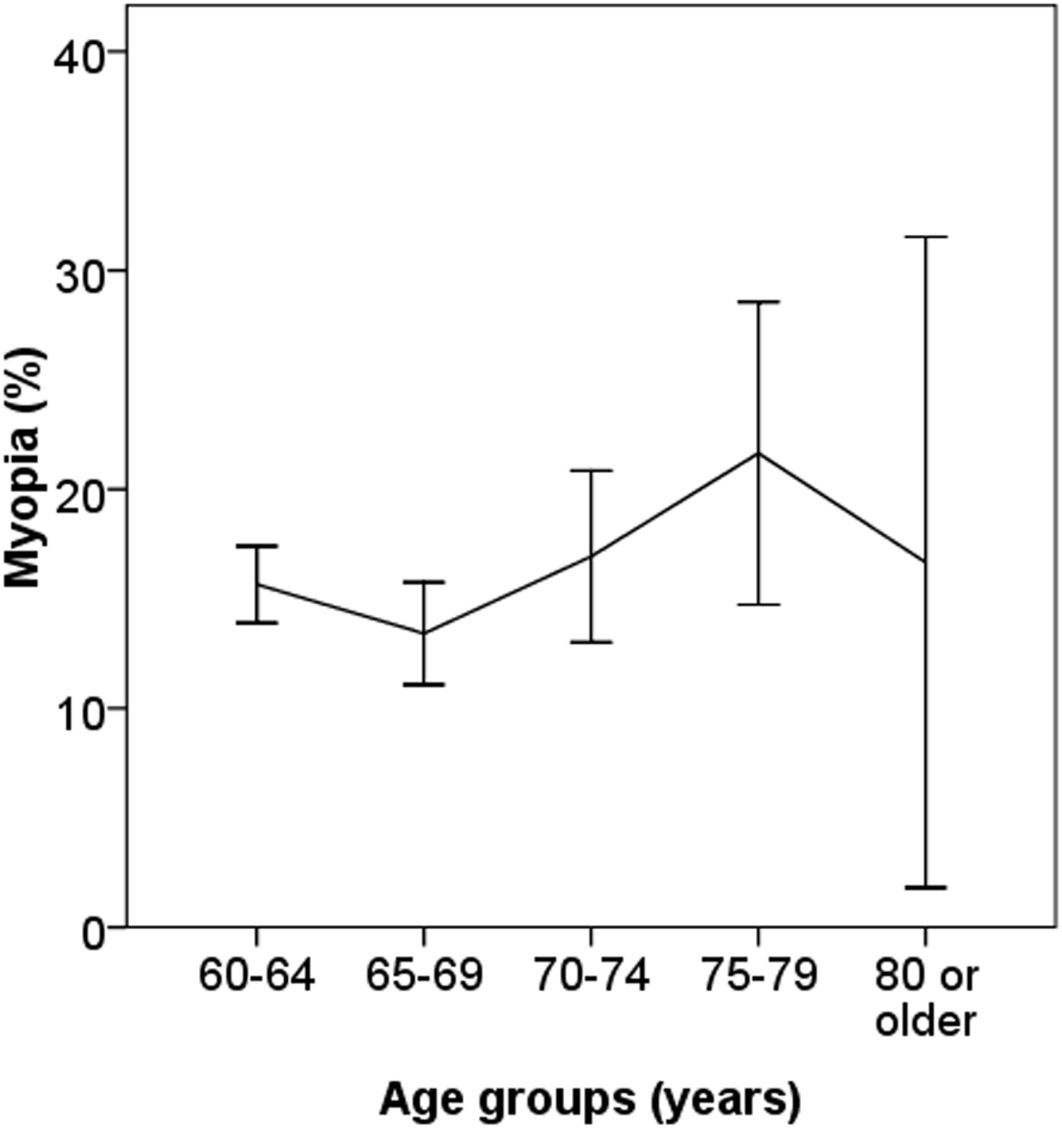
Age-specific prevalence of myopia in participants without cataract.

Of the 4611 elderly adults who participated in this study, PHQ-9 questionnaire was completed by 4597 participants. Fig 1 depicts the distribution of PHQ-9 scores among these 4597 adults. The prevalence of any depression (PHQ-9 score, 5–27) and any moderate depression (PHQ-9 score, 10–27) in this cohort was 8.0% (95CI 7.2–8.8) and 1.2% (95% 0.9–1.5). Women were more likely to have depressive symptoms compared with men (10.7% vs. 5.0%; P<0.001). Depressive symptoms were more prevalent in older age groups. For example, the prevalence of having any depressive symptoms was only 4.9% (95CI 3.9–5.8) in those aged 60 to 64 years while it was 23.4% (95CI 18.4–28.4) in those aged 80 year or older. The gender-specific prevalence of having depressive symptoms is shown in Fig 2. Among the 4597 participants who had completed the PHQ-9 questionnaire, 117 had previous cataract surgery in the right eye and were excluded from analysis. Among the remaining 4480 study participants, 22.5% and 6.8% had myopia and high myopia, respectively. The age-specific prevalence of myopia in adults without cataract is shown in Fig 3.

**Fig 4:**
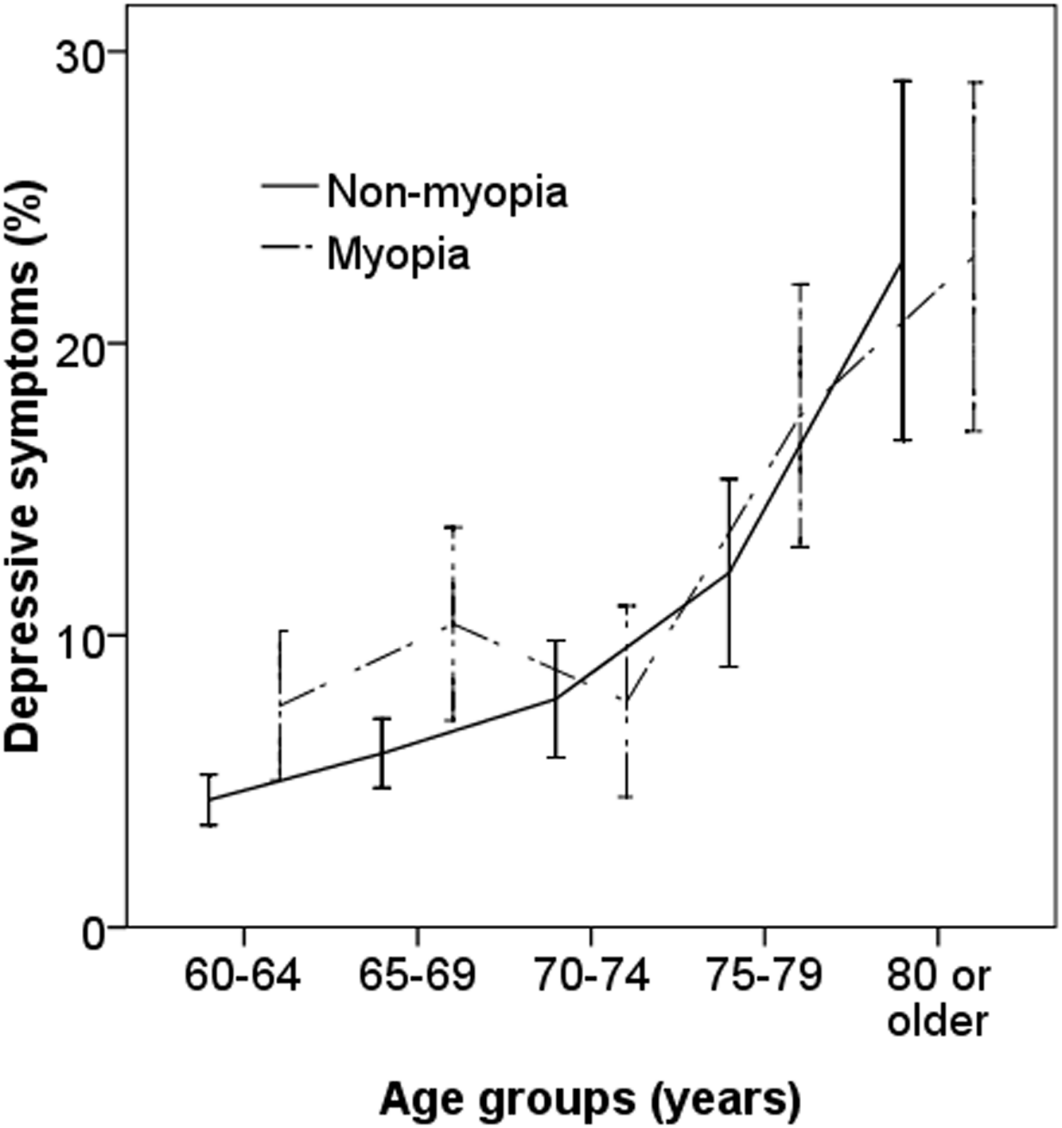
Age-specific prevalence of depression by refractive status.

Table 1 summarizes the characteristics of the study participants based on the status of having depressive symptoms as measured by PHQ-9. In this study, adults with any depressive symptoms were more likely to be older (P<0.001), female (P<0.001), living alone (P<0.001) and have diabetes (P = 0.03) compared with those without. Meanwhile, they had less monthly income (P<0.001), were less educated (P<0.001), were less likely to smoke (P<0.001), drink alcohol (P<0.001) or tea (P<0.001), spent less time outdoors (P = 0.03) spent less time watching TV (P<0.001) and slept for more hours per day (P<0.001). Myopia was more prevalent among those with depressive symptoms compared with those without (35.1% vs. 21.4%; P<0.001). The prevalence of having any depressive symptoms by refractive status (with vs. without myopia) is shown in Fig 4.

**Table 1:** Characteristics of study participants by depression status.

	Adults with depressive symptoms (n = 356)	Adults without depressive symptoms (n = 4124)	P*
Age (years)	71.3 (7.7)	67.3 (6.1)	<0.001
Female gender	255 (71.6)	2124 (51.5)	<0.001
Individual monthly income less than 1000 Yuan	106 (29.8)	485 (11.8)	<0.001
No formal education	226 (62.4)	1961 (47.6)	<0.001
Living alone	71 (19.9)	348 (8.4)	<0.001
Hypertension	222 (62.4)	2553 (61.9)	0.98
Diabetes	37 (10.4)	300 (7.3)	0.03
Smoking history	73 (20.5)	1499 (36.3)	<0.001
Alcohol intake	39 (11.0)	996 (24.2)	<0.001
Tea consumption	68 (19.1)	1503 (36.4)	<0.001
Time for watching TV per day (hours)	1.6 (1.3)	1.9 (1.4)	<0.001
Time outdoors per day (hours)	1.7 (1.6)	1.9 (1.9)	0.03
Sleeping hours per day (hours)	9.3 (1.7)	8.7 (1.4)	<0.001
Myopia	125 (35.1)	883 (21.4)	<0.001

Data presented are means (standard deviations) or number (%), as appropriate for variable.

*P value, comparing the differences between adults with and without depression, based on chi-square test or t test, as appropriate.

Table 2 demonstrates the multivariate-adjusted association of any myopia and high myopia with the presence of any depressive symptoms or moderated depressive symptoms in this cohort. After adjusting for age and gender, myopic adults had higher odds of having any depressive symptoms (OR = 1.43; 95% CI 1.10, 1.88) compared with non-myopic ones. Multivariate analysis including age, gender, myopia and factors that were significantly different in univariate comparison (P<0.05) were performed and the AIC was used to select influential explanatory variables. Any explanatory variable was excluded if it would result in a better model fit. Table 2 shows the best fitted model. This association does not change significantly if other potential confounders including education level, lifestyle-related exposures, presenting visual acuity and age-related cataract were adjusted. We found no significant differences in the risk of having any depressive symptoms between those with and without high myopia (All P > 0.05). The association of myopia or high myopia with the presence of having any moderate depressive symptoms (PHQ-9 score, 10–27) in this cohort was not significant.

**Table 2:** Relationship of myopia and high myopia with depressive symptoms.

	Age and gender adjusted model			Best fitted multivariate model*		
	OR	95%CI	P	OR	95%CI	P
Any depression						
With vs. without any myopia	**1.43**	**1.10,1.88**	**<0.001**	**1.39**	**1.04,1.92**	**0.03**
With vs. without high myopia	1.48	0.72,3.03	0.29	1.46	0.66,3.00	0.25
Any moderate depression						
With vs. without any myopia	1.45	0.73,2.88	0.30	1.40	0.70, 3.01	0.31
With vs. without high myopia	1.17	0.16,8.65	0.88	1.10	0.14,8.68	0.89

OR = odds ratio; CI = confidence interval

*Best fitted multivariate model adjusted for age, gender, educational level, time for watching TV per day, presenting visual acuity and the presence of cataract.

A significant joint effect of education with myopia on any depression was detected using a likelihood ratio test (P for interaction = 0.04). Further education-stratified analysis indicated the associations of myopia with depression was stronger in adults with no formal education (OR = 1.62) compared with those with formal education (OR = 1.31) in multivariate analysis.

## Discussion

In this community-based study of older adults aged 60 years or older, myopic adults were more likely to have depressive symptoms as measured by PHQ-9 after controlling for a range of potential confounders. The impact of myopia on depressive symptoms was found to be independent of lifestyle-related exospores, visual acuity and age-related cataract and was modified by one’s education level. These findings indicated that timely refractive assessment in older population might lead to early detection of associated mental health problems in older people.

To the best of our knowledge, this was the first study which directly assessed the impact of myopia, a common eye disorder, on depressive symptoms among older people. In the present study, the presence of myopia was significantly associated with an increased likelihood of depression in adults aged 60 years or older, and this association was even more pronounced in those without formal education. Previous reports have indicated visual impairment may be linked with depressive symptoms but the findings among different studies were inconsistent. For example, in a study of 339 older adults aged over 50 years in Armenia, those with visual impairment were more likely to have depressive symptoms compared to those without after adjusting for confounders (OR = 2.75; 95% CI: 1.29–5.87).[19] In Europeans, the prevalence of major depressive disorder and anxiety disorders were significantly higher in visually impaired older adults compared to their normally sighted peers.[20] Non-significant results were also reported. A population-based study in the United States found neither baseline best-corrected VA nor its change could predict the development of depressive symptoms.[19] Another study on American adults aged 20 to 39 years demonstrated that VA was not associated with depression disorder (P = 0.20) after adjusting for a series of confounders.[21] These inconsistent findings among different studies may be due to different study designs and characteristics of the study populations. However, visual acuity is a balanced effect of different eye disorders and if any specific eye disorders have an independent effect on depression remains unclear. Our data filled the gap of knowledge in this research area by showing that the association of myopia with depression was independent of age-related cataract, visual acuity and other potential confounders.

The biological mechanism underlying the observed associations between myopia and depression remains unclear and warrants further clarification. Our study indicated that mild but not high myopia is related to the presence of depressive symptoms. There may be some shared lifestyle-related environmental risk factors between mild myopia and depression in etiological pathways. Although we have adjusted some of the lifestyle-related factors such as time spent outdoors and time for watching TV per day, other factors not captured in this study may also meditate the association between myopia and depressive symptoms.

Another interesting finding was that the impact of myopia on depressive symptoms was stronger in people without formal education as compared with the ones who were formally educated. Adults with different education levels may need to cope with various psychosocial issues due to disparities in lifestyles, responsibilities, or circumstances. On the other hand, perceived costs of attending an eye care clinic and the lack of knowledge of the potential benefits from myopia prevention might also inhibit many poorly educated ones from seeking medical assistance. This interaction effect observed in this study suggests a complex interplay between eye disorders, socioeconomic status and people’s mental health, which requires further investigation.

Although this study had several strengths, including a large and representative sample and the use of a well-validated instrument to measure depressive symptoms, there were still some limitations, which need to be acknowledged. The number of people with moderate depressive symptoms was small in this population. We were unable to detect a significant association between myopia and moderate depressive symptoms, probably due to the lack of statistical power. This also applied to the association between high myopia and depression. The main differences in depressive symptoms between adults with and without myopia is in the younger age group (60–70 years old), and this probably accounts for most of the association observed in this study. In addition, as cultures may have a great impact on people’s mental health, the findings from Chinese population may not be directly extrapolated to other ethnic groups considering the huge disparities in cultures among different ethnic groups. Furthermore, although we have adjusted for a wide range of confounders in multivariate analysis, residual confounding may still exist. For example, cataract may affect the detection of refractive error, especially for older people. The effect of cataract may not have been completely removed even though it was adjusted in multivariate analysis. Finally, we used ORs as the effect estimates when measuring the association between myopia and depressive symptoms. The use of ORs might overestimate the relative risk of myopia for depressive symptoms.

In conclusion, we found a significant association between myopia and the presence of depressive symptoms among older Chinese adults, particularly in poorly educated ones. Ophthalmologists and healthcare practitioners should be aware of these findings so as to improve the health-related quality of life among myopic individuals by recognizing the signs and symptoms for depression and referring them for mental health services in time.
